# The effect of Lameness before and during the breeding season on fertility in 10 pasture-based Irish dairy herds

**DOI:** 10.1186/s13620-015-0043-4

**Published:** 2015-06-23

**Authors:** Joris R. Somers, Jon Huxley, Ingrid Lorenz, Michael L. Doherty, Luke O’Grady

**Affiliations:** School of Veterinary Medicine, University College Dublin, Belfield, Dublin 4, Ireland; School of Veterinary Medicine and Science, University of Nottingham, Sutton Bonington Campus, Sutton Bonington, Leicestershire, LE12 5RD UK

**Keywords:** Lameness, Dairy, Cows, Pasture, Fertility, Locomotion score, Breeding season, Pregnancy rate

## Abstract

**Background:**

The effects of lameness on fertility have been documented frequently but few data are available from seasonally breeding, pasture-based herds (such as those used in Ireland) where cows are housed during the winter months but managed at pasture for the remainder of the year. This study determined the prevalence of lameness in a group of 786 cows in 10 pasture-based Irish dairy herds before, during and after the breeding season and assessed the relationship between lameness and the reproductive performance in these herds through serial locomotion scoring during the grazing period.

**Results:**

Lameness prevalences of 11.6 % before, 14.6 % during and 11.6 % after the breeding season were found and these compared favourably to results from housed cattle and are similar to other studies carried out in grazing herds. A Cox proportional hazards model with locomotion score as time varying covariate was used. After controlling for the effect of farm, month of calving, body condition score at calving, body condition score loss after calving and economic breeding index, cows identified as lame during the study were less likely to become pregnant. Cows lame before the earliest serve date but no longer lame during the breeding season, cows becoming lame after the earliest serve date and cows identified lame both before and after this date were respectively 12 %, 35 % and 38 % less likely to become pregnant compared to cows never observed lame during the study. However, these findings were only significant for cows becoming lame after the earliest serve date and cows lame both before and after the start of breeding.

**Conclusions:**

This study found that the reproductive efficiency was significantly (p < 0.05) lower in cows becoming lame during the breeding season and cows lame before and during the breeding season compared to non-lame cows. Cows no longer lame during the breeding season had a lower Submission Rate to first serve within 3 weeks of earliest serve date. However, the Pregnancy Rate was not significantly (p > 0.05) lower in these animals compared to cows never diagnosed as lame. In addition to lameness status, nutritional status and genetics were found to influence the reproductive performance in pasture-based Irish dairy herds.

## Background

Together with mastitis and poor fertility, lameness in cattle is one of three major factors influencing profitability and economic losses in modern dairy farming [1, 2]. In contrast to infertility and sub-clinical mastitis, lameness has a distinct negative effect on animal behaviour and welfare [3–5]. Lameness in dairy cows is a worldwide problem with herd prevalence estimates ranging from 8 % in New Zealand [6], 22 % in Chile [7], 32 to 37 % in the UK [4, 8, 9] to 55 % in North America [10]. In general, higher levels of lameness are reported in dairy herds managed under zero-grazing systems compared to grazing herds [11]. In housed cattle cow comfort plays an integral role in the onset and prolongation of lameness [12, 13]. Under grazing conditions, distance walked and the condition of the roadways has the biggest influence on disease development [14, 15]. Most studies on lameness are based on these two very distinct management systems, less data are available from seasonally breeding, pasture-based herds (such as those used in Ireland) where cows are housed during the winter months but managed at pasture for the remainder of the year.

The effects of lameness on fertility are extensively documented. Numerous controlled studies have found that calving to first service interval and calving to conception interval were extended in lame cattle [12, 16–19]. However, the post-partum resumption of cyclicity was not delayed by the presence of lameness in a study by Aungier et al. [20] or only delayed in cows diagnosed with severe lameness [21]. Additioinally, Barkema et al. [18] found that lameness prolonged the interval between first service and conception by 3.4 days. Many studies reported lower conception rates in lame cows [12, 17, 22] and reduced intensity of oestrus may be a cause of poor reproductive performance in these animals [3, 16, 23].

During the relatively short breeding season, reproductive efficiency is of great importance if key reproductive targets are to be achieved. A multitude of factors can affect the extent to which these reproductive targets will be met during the breeding season [20]. Lameness is one of these factors, often mentioned in relation to decreased fertility.

The objective of this study was [1] to determine the prevalence of lameness in a group of 10 Irish dairy herds and [2] to assess the relationship between lameness and the reproductive performance in pasture-based dairy herds through serial locomotion scoring at various stages of the breeding season.

## Methods

### Farm and animal selection

This prospective observational study was carried out in 2013 on 10 commercial Irish dairy farms located in Co. Kildare and Co. Wicklow. The predominant breed on these farms was Holstein-Friesian. These 10 farms were part of an on-going herd health management programme conducted by University College Dublin (UCD). All the farms used seasonal breeding and were visited by a UCD veterinarian every 21 days during the calving and breeding season. During these visits the main focus was on the fertility performance and the nutritional management of the cows. Concentrate supplementation in the milking parlour was applied by all 10 farms during times of the grazing season when energy intake from grass alone was not enough to fulfil the cow’s energy requirements for maintenance and production.

Data recorded as part of the herd health programme were:

- Monthly milk recording including yield to date, predicted 305 day energy corrected milk (ECM) yield, somatic cell count and milk constituents
- Economic breeding index (EBI); a single figure profit index aimed at identifying the most genetically profitable bulls and cows for breeding replacements [24].
- Calving date, calving difficulty and peri-parturient disease events
- Body condition score (BCS) [25] at calving, pre-breeding, services, pregnancy diagnoses and drying off
- Ultrasound-based pre-breeding examination, pregnancy diagnosis at 30 and 60 days after insemination and anoestrous examination

Only cows calved between 1^st^ January and 21^st^ May, 2013 and declared for breeding in the spring breeding herd were included in the study. The spring breeding herd size on the 10 farms ranged from 40 to 140 cows. The breeding season occurred between 25^th^ March and 16^th^ August and varied between 84 and 130 days, depending on farm. The voluntary waiting period (VWP) for all 10 farms was 42 days in milk (DIM) and a combination of artificial insemination (AI) and bulls were used during the breeding season.

### Reproductive management

From the end of February, the lactating cows were at pasture both day and night. The late gestation dry cows remained housed until they calved at which stage they were turned out to pasture immediately. In the Irish seasonal breeding system, cows are only eligible for breeding from the mating start date (MSD), upon completion of the VWP. The first day a cow is eligible for breeding is the earliest serve date (ESD). The breeding window (BW) in which cows could possibly be bred was a median of 13 weeks (range: 4 – 19 weeks) in the present study population.

Direct observation of oestrus was used for heat detection by the farmers. Tail paint, vasectomised bulls or pedometers were used as heat detection aides. Based on oestrus events, animals were submitted for AI by the farmer or AI technicians. Natural bull serves were also recorded by the farmer, either as observed events or retrospectively based on foetal age estimation at pregnancy diagnosis.

Reproductive performance was monitored at a herd-level based on Submission rate (SR) to first serve within 3 weeks from ESD, Conception risk (CR) to first serve and overall CR and Pregnancy rate (PR). PR is the incidence rate at which eligible cows become pregnant. Survival analysis is the appropriate way of presenting PR as this also accounts for censoring. Both SR, as the time to service, and CR, as the risk of success, determine PR, making this the most comprehensive parameter to assess herd reproductive performance [26].

### Lameness recording and management

Lameness data were gathered by means of serial locomotion scoring of all the animals in the breeding herd. Between 25^th^ February and 20^th^ August, all 10 farms were visited 4 times by the same UCD veterinarian. Visits were scheduled in accordance with the breeding season to allow for cows to be locomotion scored once before the start of the breeding season, twice during the breeding season and once when the breeding season was finished. Cows were identified by freeze brand number and locomotion scored by a single observer (JS) as they exited the milking parlour after morning milking using the 5-point scale described by Sprecher et al. [17]. Farmers were given a list of lame cows at the end of each scoring session. Cows on this list were submitted for treatment by the same professional foot trimmer for all 10 farms within 8 days of the scoring session.

### Data management

The data gathered as part of the herd health programme were recorded on the farming software package Herd Master (Irish Farm Computers Ltd.) These data were exported to a Microsoft Office Excel 2010 (Microsoft Corp. Redmond, WA) spreadsheet for calculating the reproductive parameters and evaluating the reproductive performance of the herds at the end of the breeding season. The data obtained during the locomotion scoring visits were added to this spreadsheet.

Animals were categorised into 2 lactation groups representing 1^st^ lactation cows (n = 250) in 1 group and 2^nd^ to 10^th^ lactation cows (n = 536) in the other group. Cows calved in the months January and February were grouped as were cows calved in April and May. A case of lameness was defined as an animal that received a locomotion score (LS) ≥ 3 on the 1 – 5 scale at least once during the course of the study. Each case of lameness was further identified as being observed lame before and/or after the animal’s ESD. This resulted in 4 categories of animals in the population, those not observed lame before or after ESD (NN), those observed lame before but not after ESD (YN), those observed not lame before but lame after ESD (NY) and those observed lame before as well as after ESD (YY).

Statistical analysis was carried out using the ‘survival’ package [27] in R (R Development Core Team 2014). Descriptive analysis was performed on farm and cow data. The effect of lameness on the reproductive performance, measured as PR, was analysed using the number of days for cows to become pregnant after ESD for each of the different categories of timing of detection of lameness. Kaplan-Meier survival curves are a convenient method to summarise time-to-event data [28]. Survival estimate curves were used to compare the PR distribution between NN, YN, NY and YY cows and to calculate median days to conception (Table 3). The 95 % CI were calculated based on log (survival) for each group. and a log-rank test was performed to compare overall survival of the 4 groups of cows [28]. A Cox proportional hazards model with lameness as time varying covariate [28, 29] was used to identify variables influencing the PR. Right-censoring occurred for animals that left the herd or did not conceive during the breeding period. Explanatory variables were screened by entering each into a univariate Cox proportional hazards model. Significance testing was performed using the Wald *χ*^*2*^-test and those variables with P-value ≤0.25 were retained for inclusion in a multivariable Cox proportional hazards model. Farm was included in the analysis as cluster effect. Explanatory variables were removed in a backwards elimination process using P-value >0.05 as reason for removal. One exception was that lameness status was included in all multivariate models, because the intent was to determine the relationship between lameness status and PR while controlling for other important factors. Additionally, two-way interactions were assessed and retained if they were significant at an α-value of ≤0.05. A variable was considered as a confounder if it influenced other covariates estimates by >20 % [28]. The daily hazard of pregnancy [*h*_*i*_(*t*)] was calculated as

$$ {h}_i(t) = {h}_0(t) \exp \left[\left({\beta}_1{\chi}_{1i} + \dots + {\beta}_m{\chi}_{mi}\right) + {\gamma}_1{z}_1(t)\right] $$

where *h*_*0*_(*t*) represents the baseline hazard as a function of time; *β1, . . . , βm* represent the regression coefficients for each of the m time-invariant covariates (month of calving, BCS at calving, maximum BCS lost after calving, and EBI overall). *X*_*1i*_*, . . . , X*_*mi*_ represent the covariates for each of the observations; *γ*_*1*_ is the estimated regression coefficient for effect of lameness as a time varying covariate on day (*t*); and *z*_*1*_ is lameness status (0, 1) at day (*t*).

The proportional hazards assumption of the Cox model was tested for categorical variables by examining for each stratum plots of log[−logS(*t*)], where S(*t*) is the survival function for each stratum, against time with the expectation that the plots would be parallel. For continuous variables, the proportional hazard assumption was tested using a plot of the scaled Schoenfeld residuals as a function of time to event with the expectation that no association would occur between the residuals and time to event.

The scale of each continuous variable was tested using the Martingale residuals. A plot of Martingale residuals from a model that excludes the continuous variable against the values of the excluded continuous variable was examined with the assumption that the relationship would be linear.

## Results

### Population Descriptors

Table 1 provides descriptive details on the study population. A total of 786 cows were included in the 2013 spring breeding population across the ten farms. 86.1 % calved between January 1^st^ and March 31^st^. The remaining 13.9 % calved during April and May. First lactation animals made up 31.8 % of the population. Reproductive performance measures were calculated. SR to 1^st^ serve within 3 weeks of ESD was 82.8 %, CR to 1^st^ serve was 47.7 %, overall CR was 49.7 % and at the end of the breeding season, 80.7 % of the population was pregnant. The 24-day PR was 51.3 %.

**Table 1:** Study population descriptive statistics of parity, calving date, milk production, BCS at calving and BCS loss post calving, EBI, breeding season characteristics and the number of locomotion scores recorded per cow during the study

Variable	N	Mean (SD)	Median	Min / Max^a^
Parity	786	2.9 (1.4)	2	1 / 10
1^st^ Lactation	250			
≥2^nd^ Lactation	536			
Calving date		26/2/13 (26.7 days)	20/2/13	1/1/13 / 21/5/13
305 day ECM yield (Kg)		6688.0 (1412.6)	6704	3387 / 11720
Fat and Protein produced (Kg)		493.3 (104.1)	491	250 / 883
BCS at calving		3.1 (0.2)	3	3 / 4
Max^a^ BCS loss post calving		0.3 (0.2)	0	0 / 1
DIM to max loss (days)		55.8 (34.2)	45	10 / 246
EBI overall (€)		125.4 (43.6)	130	−77 / 249
EBI fertility (€)		62.8 (32.5)	66	−48 / 148
ESD		26/4/13 (14.7 days)	24/4/13	25/3/13 / 2/7/13
DIM at ESD (days)		58.4 (16.7)	56	29 / 115
Breeding season length (weeks)		13.4 (2.2)	13	4 / 19
Eligible for breeding (days)		38.2 (32.9)	27	1 / 130
Locomotion scoring session 1 to ESD interval (days)		33.9 (23.5)	36	−54 / 120
No. of LS recorded		3.8 (0.5)	4	1 / 4

^a^Min = minimum; Max = maximum

### Locomotion Scoring

Descriptive locomotion scoring results are shown in Table 2. At least one LS was recorded for 786 cows and the mean number of locomotion scores recorded per cow was 3.8 (SD: 0.5) (Table 1). LS 3 or higher was awarded to a total of 180 cows. 771 cows had LS recorded both before and after ESD (Table 3) and were included in the cox proportional hazard model. 77.1 % of the herd was never observed with LS greater than 2 during the study.

**Table 2:** Descriptive statistics of the lameness detection in relation to the breeding season, based on 1 locomotion scoring session before ESD and 3 sessions after ESD of which 2 session occurred during the breeding season and 1 session after the breeding season had already finished

Detection of LS ≥ 3	N	Population size	Prevalence %
Overall during study	180	786	22.9
Before ESD	90	773	11.6
During breeding	115	785	14.6
After breeding	90	776	11.6

**Table 3:** Cows for which LS was recorded before and after ESD were classified into 1 of 4 categories: NN for cows never detected lame, YN for cows lame before ESD and LS <3 after ESD, NY for cows LS <3 before ESD and lame after ESD and YY for cows lame before and after ESD. SR, overall CR, number of serves per conception and Kaplan-Meier median survival time is shown for each lameness detection category

LS ≥ 3 detection categories	N (%)	SR (%)	CR (%)	serves/conception	K-M median survival time (95 % CI)
NN	612 (79.4)	85.2	50.3	2.0	26 (24–30)
YN	47 (6.1)	66.0	59.1	1.7	33 (25–48)
NY	69 (8.9)	81.2	43.4	2.3	36 (24–70)
YY	43 (5.6)	72.1	41.4	2.4	43 (28–74)

### Detection of lameness in relation to ESD

The effect of detecting lameness before or after ESD on the PR is shown in Table 4. The only variables retained in the model were lameness detection group, BCS at calving, BCS loss after calving, month of calving group and EBI. There were no significant interactions in the model. After controlling for the effect of BCS at calving, BCS loss after calving, month of calving and EBI, the hazards for pregnancy decreased by a factor 0.88 (CI: 0.62 – 1.29), 0.65 (CI: 0.52 – 0.81) and 0.62 (CI: 0.42 – 0.93) for YN cows, NY cows and YY cows respectively compared to NN cows. Cows with BCS 3 and 3.25 at calving had increased hazards for pregnancy (1.28 CI: 1.01 – 1.64 and 1.55 CI: 1.15 – 2.07 respectively) compared to cows with BCS <3 at calving. Cows with BCS at calving ≥3.5 did not have a significantly different hazard ratio (HR) for pregnancy compared to cows with BCS <3 at calving. Cows losing BCS after calving also had a lower hazard for pregnancy compared to cows that did not lose BCS after calving. BCS loss data analysis resulted in HR 0.66 (CI: 0.52 – 0.85) for cows losing 0.25 BCS after calving, 0.59 (CI: 0.44 – 0.78) for cows losing 0.5 BCS after calving and 0.37 (0.23 – 0.57) for cows losing >0.5 BCS after calving. Cows calved later in the year had a significantly decreased hazard for pregnancy. Increased EBI overall resulted in an increased hazard for pregnancy.

**Table 4:** Multivariate Cox proportional hazards model for pregnancy rate using detection of lameness before and after ESD as time dependent variables. Farm is included as a cluster effect in the analysis. There were no significant interactions in the model

Variable	N	Coefficient (SE)	P-value	Hazard ratio (95 % CI)
Lameness detection group				
NN	612			1.00
YN	47	−0.13 (0.17)	>0.05	0.88 (0.62 – 1.29)
NY	69	−0.42 (0.15)	<0.01	0.65 (0.52 – 0.81)
YY	43	−0.46 (0.19)	<0.05	0.62 (0.42 – 0.93)
BCS at Calving				
≤2.75	162			1.00
3.0	314	0.25 (0.13)	<0.05	1.28 (1.01 – 1.64)
3.25	243	0.44 (0.15)	<0.01	1.55 (1.15 – 2.07)
≥3.5	52	0.41 (0.23)	>0.05	1.51 (0.96 – 2.36)
Max BCS loss after Calving				
0	143			1.00
0.25	315	−0.41 (0.12)	<0.01	0.66 (0.52 – 0.85)
0.5	252	−0.53 (0.14)	<0.01	0.59 (0.44 – 0.78)
>0.5	61	−1.0 (0.23)	<0.01	0.37 (0.23 – 0.57)
Month of Calving				
Jan/Feb	457			1.00
March	206	−0.33 (0.10)	<0.01	0.72 (0.60 – 0.86)
Apr/May	108	−0.69 (0.15)	<0.01	0.50 (0.37 – 0.68)
EBI overall (€)	771	0.01 (0.001)	<0.01	1.00 (1.00 – 1.01)

## Discussion

The 11.6 %, 14.6 % and 11.6 % point prevalence of lameness found during the grazing season in the present study compared favourably to 39 % found in housed cattle [11] and was similar to 8 % and 15 % found in grazing herds [6, 11]. 22.9 % of the herd was identified as lame at least once during the course of the study. The proportion of animals already lame before the study start could not be determined. The 8.9 % cows becoming lame during the breeding season is probably a more realistic representation of lameness incidence risk and is lower than the estimated incidence risk of 15 % found by Alawneh et al. [12]. However, the frequency of locomotion scoring visits to the individual farms involved in this study was too low to allow for precise lameness incidence risk calculation. Combining LS 3, 4 and 5 cows into the group of lame cows provided significant evidence of the negative effects of lameness on fertility. However, including LS 2 cows in the group of lame cows did not show an effect on fertility [20]. Many papers have been published on the effect of lameness on fertility in dairy cattle. However, there is dearth of information on how both the level of lameness and the timing of onset of lameness influence the reproductive performance under seasonally breeding conditions. The results of this study show that animals becoming lame during the breeding season and those found lame before and during the breeding season are significantly less likely to become pregnant compared to cows never detected lame or cows no longer detected as lame after ESD. This reduced reproductive performance can have an effect on the subsequent lactation and, thus the economic productivity of the cow [12]. In the study carried out by Alawneh et al. [12], the daily CR for lame cows was decreased by a factor of 0.78 compared to non-lame cows. This same study also found that the interval between the planned start of mating and conception was extended by 12 days in lame cows compared to non-lame cows. Collick et al. [16] reported a CR to first serve for lame cows of 46 % while this was 56 % for non-lame cattle during winter housing. In the present study, logistic regression analysis of lameness status on CR was performed in a separate analysis (results not shown) and showed no significant effect of lameness on CR (p > 0.05). As a result, the effect of lameness on PR must be attributed to the effect of lameness on SR. This is supported by the conclusion of Barkema et al. [18] that lameness did not affect CR to first serve. As a painful disorder, lameness impedes cows to express oestrus, making them less likely to be inseminated [16, 17, 19, 26]. Univariate analysis of our data shows a reduced SR to first serve within 3 weeks of ESD for cows diagnosed as lame before ESD. Cows recovering from this episode of lameness are mathematically less likely to become pregnant compared to cows never diagnosed as lame, but this lower HR was not significant (p > 0.05). The cause and effect relationship between lameness and decreased reproductive performance, found in the present study, could not be further explored. Melendez et al. [22] found associations between lameness and higher incidence of ovarian cysts and delayed resumption of cyclicity post-partum. But this was contradicted in a more recent study [20] that did not find a significant effect of lameness on reproductive target achievement in the post-partum period. Aggravated negative energy balance, expressed as excessive BCS loss after calving, was found to increase the probability of delayed resumption of cyclicity in both studies [20, 22]. Low BCS at calving and BCS loss post-partum have been associated with the development of lameness and reduced recovery from lameness [30, 31]. In the current study, the interaction between low BCS and lameness was not explored beyond the statistical model’s two-way interactions assessment. Whether impaired ovarian function or decreased oestrus expression caused reduced SR in lame cows could not be established.

The current study does have limitations. Firstly, our data were from a small group of 10 farms involved in a herd health programme. The relationship between lameness and reproductive performance is inevitably influenced by a farm effect. Farms where cases of lameness are identified and managed effectively are less likely to have decreased reproductive performance due to lameness than farms where lameness is insufficiently managed. Lameness management was different between the 10 study farms [32] and can often be used as an overall indication of animal husbandry management on the farm. Furthermore, lame animals were treated by a professional foot trimmer shortly after being detected as a case of lameness, decreasing the number of potentially chronically lame animals in the herd. Foot lesions were recorded during foot trimming, but analysis of the causes of lameness was not included in this study. Infectious causes of lameness with a short incubation period and a rapid response to treatment probably have a lower effect on fertility than claw horn lesions, which have a much longer pathogenesis and respond more slowly to treatment [12].

## Conclusions

Reproductive efficiency is of even greater importance in seasonal breeding systems compared to conventional dairy management systems. This study found that the PR, which represents reproductive efficiency, was significantly lower in cows becoming lame during the breeding season and cows lame before and during the breeding season compared to non-lame cows. Cows no longer lame during the breeding season had a lower SR to first serve within 3 weeks of ESD. However, the PR was not significantly lower in these animals compared to cows never diagnosed as lame. In addition to lameness status, BCS at calving, BCS loss after calving, month of calving and EBI were found to influence PR in pasture-based dairy herds. Apart from lameness being a reason for culling, lame cows are more likely to be culled after the breeding season due to poor fertility, aggravating the economic impact of lameness in seasonal breeding systems [5, 12, 17, 26, 33, 34]. Further analysis of lameness data, incorporating production and energy balance data, is required to further understand the dynamics of lameness in pasture based, seasonally breeding dairy herds.

## Abbreviations

UCD: University College Dublin
ECM: Energy Corrected Milk
EBI: Economic Breeding Index
BCS: Body Condition Score
VWP: Voluntary Waiting Period
DIM: Days in Milk
MSD: Mating Start Date
ESD: Earliest Serve Date
BW: Breeding Window
AI: Artificial Insemination
SR: Submission rate
CR: Conception risk
PR: Pregnancy rate
LS: Locomotion Score
NN: Cows not lame before or after ESD
YN: Cows lame before but not lame after ESD
NY: Cows not lame before but lame after ESD
YY: Cows lame before and after ESD
HR: Hazard ratio
